# High Rates of HIV Seroconversion in Pregnant Women and Low Reported Levels of HIV Testing among Male Partners in Southern Mozambique: Results from a Mixed Methods Study

**DOI:** 10.1371/journal.pone.0115014

**Published:** 2014-12-26

**Authors:** Caroline De Schacht, Heather J. Hoffman, Nédio Mabunda, Carlota Lucas, Catharina L. Alons, Ana Madonela, Adolfo Vubil, Orlando C. Ferreira, Nurbai Calú, Iolanda S. Santos, Ilesh V. Jani, Laura Guay

**Affiliations:** 1 Elizabeth Glaser Pediatric AIDS Foundation, Research Department, Maputo, Mozambique; 2 The George Washington University, Milken Institute School of Public Health, Department of Epidemiology and Biostatistics, Washington, D.C., United States of America; 3 Instituto Nacional de Saúde, Maputo, Mozambique; 4 Federal University of Rio de Janeiro, Biology Institute, Rio de Janeiro, Brazil; 5 Ministry of Health, Xai-Xai, Mozambique; 6 Ministry of Health, Matola, Mozambique; 7 Elizabeth Glaser Pediatric AIDS Foundation, Research Department, Washington, D.C., United States of America; University of Washington, United States of America

## Abstract

**Introduction:**

Prevention of acute HIV infections in pregnancy is required to achieve elimination of pediatric HIV. Identification and support for HIV negative pregnant women and their partners, particularly serodiscordant couples, are critical. A mixed method study done in Southern Mozambique estimated HIV incidence during pregnancy, associated risk factors and factors influencing partner's HIV testing.

**Methods:**

Between April 2008 and November 2011, a prospective cohort of 1230 HIV negative pregnant women was followed during pregnancy. A structured questionnaire, HIV testing, and collection of dried blood spots were done at 2–3 scheduled visits. HIV incidence rates were calculated by repeat HIV testing and risk factors assessed by Poisson regression. A qualitative study including 37 individual interviews with men, women, and nurses and 11 focus group discussions (n = 94) with men, women and grandmothers explored motivators and barriers to uptake of male HIV testing.

**Results:**

HIV incidence rate was estimated at 4.28/100 women-years (95%CI: 2.33–7.16). Significant risk factors for HIV acquisition were early sexual debut (RR 3.79, 95%CI: 1.04–13.78, p = 0.04) and living in Maputo Province (RR 4.35, 95%CI: 0.97–19.45, p = 0.05). Nineteen percent of women reported that their partner had tested for HIV (93% knew the result with 8/213 indicating an HIV positive partner), 56% said their partner had not tested and 19% did not know their partner test status. Of the 14 seroconversions, only one reported being in a serodiscordant relationship. Fear of discrimination or stigma was reported as a key barrier to male HIV testing, while knowing the importance of getting tested and receiving care was the main motivator.

**Conclusions:**

HIV incidence during pregnancy is high in Southern Mozambique, but knowledge of partners' HIV status remains low. Knowledge of both partners' HIV status is critical for maximal effectiveness of prevention and treatment services to reach elimination of pediatric HIV/AIDS.

## Introduction

Prevention of mother-to-child transmission (PMTCT) of HIV includes prevention of incident infection among women of reproductive age, especially during pregnancy [1]. Women during pregnancy and the postpartum period have an increased risk of acquiring HIV, similar to high-risk sub-populations, such as female sex workers, men having sex with men and known serodiscordant couples [2]. Therefore, strong preventive services, including identification of serodiscordancy among pregnant couples is crucial to decrease HIV transmission.

Most PMTCT programs have achieved high rates of initial HIV testing, with periodic repeat testing recommended for HIV negative pregnant women. However, partner involvement in mother and child health (MCH) services is mostly restricted to requests for HIV testing during antenatal care [3] with poor uptake in sub-Saharan Africa [4]–[7].

Mozambique has a high HIV burden, with a national prevalence of 16% among pregnant women and the highest prevalence (24%) occurring in Southern Mozambique [8]. The national PMTCT program includes opt-out testing for pregnant women, with all women encouraged to invite their partners for HIV testing at the clinic. In the 2009 national survey on HIV prevalence, knowledge and risk behaviors, analysis of HIV testing among couples revealed that 64% reported neither partner had tested, 20% reported only the female partner was tested, 5% only the male partner tested and 11% reported both partners were tested [9]. Reasons for the low uptake of male partner testing were not assessed.

The Elizabeth Glaser Pediatric AIDS Foundation and the Instituto Nacional de Saúde (INS) undertook a study to estimate HIV seroconversion (defined by repeat HIV rapid testing) and its associated factors in pregnant women in Southern Mozambique. Knowing the HIV status of the male partner is an important factor in decreasing the risk of incident infection during pregnancy and beyond by providing appropriate preventive services. This factor, combined with the low rates of partner testing, highlighted the need for a qualitative component to be added to the study, allowing for the exploration of motivators and barriers to the uptake of HIV testing by male partners.

## Methods

The study was conducted in six rural health facilities and their surrounding communities in Gaza (Chibuto, Chicumbane and Malehice) and Maputo (Marracuene, Moamba and Boane) provinces in Southern Mozambique. Facilities were chosen by convenience because of high HIV prevalence and antenatal care (ANC) attendance. The study used a mixed methodology with a prospective cohort of HIV negative pregnant women attending ANC services followed during pregnancy and a qualitative formative evaluation involving men, women, grandmothers and health care workers (HCW) from the health facilities and surrounding communities. Data collection was done between April 2008 and November 2011. After delivery, women were eligible to enroll in a separate postpartum incidence cohort [10].

### Prospective Cohort Study

HIV negative pregnant women attending their first or subsequent ANC visits were invited to participate in the cohort by a trained MCH nurse. Screening and enrollment were done consecutively and concurrently in the six study sites until the desired sample size was reached. Inclusion criteria were age ≥18 years, gestational age ≤32 weeks, and a negative HIV test at enrollment. A trained study counselor administered a structured questionnaire during three scheduled study visits at enrollment, 28 weeks of gestational age, and delivery. The study visits were scheduled during women's routine antenatal care visits, while the study visit at delivery usually happened soon after delivery. Women who enrolled between 28 and 32 weeks of gestational age participated in only two of the three study visits (enrollment visit and delivery). Information on sexual activity, condom use, and knowledge of partner serostatus was collected. The enrollment questionnaire included additional questions on socio-demographic data and knowledge of HIV/PMTCT. HIV rapid testing and collection of dried blood spots (DBS) were done by the MCH nurse at each visit.

On site HIV testing was performed as per the national guidelines using a sequential algorithm of two rapid tests. Screening was conducted using the Determine HIV1/2 (Abbott Laboratories, Wiesbaden, Germany) rapid assay. Women testing non-reactive on this first test were regarded as HIV-negative while those with reactive results were further tested using the UniGold Recombigen (Trinity Biotech, Co Wicklow, Ireland). Women with reactive results on both assays were considered to be HIV-infected. Women with discordant rapid test results were requested to return four weeks later for re-testing, as per national guidelines. Testing procedures were conducted as per the manufacturer's instructions for whole blood specimens. External quality assessment of rapid testing was performed by the reference laboratory as part of the National Quality Assurance Program, with proficiency panels being sent twice a year.

Women who seroconverted during the study were provided with follow up care as per the national guidelines. Confirmatory testing for women with HIV seroconversion was conducted on DBS samples from the last and all previous visits using HIV DNA-PCR analysis (Roche Amplicor HIV-1 DNA Test, version 1.5, Roche Molecular Diagnostics, Branchburg NJ). All confirmatory testing and storage of samples was done in the INS national immunology reference laboratory in Maputo.

Based on previous studies, we assumed that the percentage of women seroconverting during pregnancy and postpartum period is approximately 12% [11]. Using the large sample normal approximation, a two-sided 95% confidence interval with 2% precision for a maximum expected proportion of 0.12 requires a sample size of 1015 pregnant women. Allowing for 10% loss to follow-up, sample size was calculated at 1128 pregnant women.

Data were double entered in an electronic database and corrected for discrepancies using EpiData Version 1.1. Statistical analysis was conducted using SAS/STAT software, Version 9.2 of the SAS System for Windows (STAT Institute Inc., Cary, NC, USA). HIV seroconversion on subsequent serological testing was used as a proxy for calculating the HIV incidence rate. For the incidence rate calculations, exposure time was calculated as the interval between time of enrollment until last HIV negative test for non-seroconverters and until the time of HIV infection for seroconverters, which was estimated as the midpoint between the last negative HIV rapid test result and the first positive HIV rapid test result. Bivariate analysis was done to estimate associations with seroincidence, using Poisson regression models. P-values below 0.05 were considered statistically significant.

### Qualitative Component

In four of the six study districts (Chibuto, Chicumbane, Marracuene, Moamba), individual interviews and focus group discussions (FGD) were conducted with fathers, paternal grandmothers, pregnant or postpartum women, and MCH nurses. Paternal grandmothers are important family decision makers in Southern Mozambique as the culture is patrilineal and many men are migrant workers.

All participants were ≥18 years of age and had to be able to provide written informed consent. The study population criteria included being a father; a paternal grandmother of a child <2 years old, a pregnant woman or mother of a child <2 years old; or an MCH nurse.

The interviews were done with women attending the MCH clinic, men attending the outpatient clinic and MCH nurses in the four study health facilities. After receiving information about the study, health care workers in antenatal, postnatal and outpatient clinics identified potential study participants among men and women attending these services. Clients received information about the study during the daily health information session and if interested, were referred to the study team who screened for eligibility. Pregnant women did not have to part of the cohort study to participate in the qualitative component. For the health care workers interviews, MCH nurses who were available on the day of data collection were invited by study staff to participate. All interviews were held in a private room at the health facility.

FGD were conducted with grandmothers, men, and women living in the surrounding communities of the health facilities. The study team approached community based organizations or community leaders in these areas and informed them of the study. They identified and informed possible participants of the study and coordinated the meeting for the focus group at a time and place convenient for the participants. Investigators used structured interview and FGD guides consisting of questions on knowledge, perceptions, attitudes and behavior regarding HIV testing for partners of pregnant women, either within MCH services or other services at the health facility. Vignettes with hypothetical scenarios were used during the discussions.

The interviews and FGD were conducted in Portuguese or Changana. Audiotapes and notes were translated to Portuguese and entered in Microsoft Word. Transcripts were manually coded by two researchers independently. The results were compared and discrepancies were discussed until consensus was reached. Analysis was done using MAXqda Version 10 (Verbi GmbH, Berlin, Germany). Deductive codes originated from the research questions, and inductive codes emerged from the data. Data displays with coded responses by participant group for each code were developed and compared. Data reduction was conducted through detailed matrices on each theme [12]. Descriptive analysis of participants' demographic data was done with STATA Version SE/11.0 (StataCorp, Texas, USA).

The Mozambique National Health Bioethics Committee (*CNBS, Comité Nacional de Bioética para Saúde*) in Mozambique reviewed and approved the study. Study staff obtained written informed consent from all participants in Portuguese or in the local language (Changana) before enrollment.

## Results

### Characteristics of the study population

**Figure 1:**
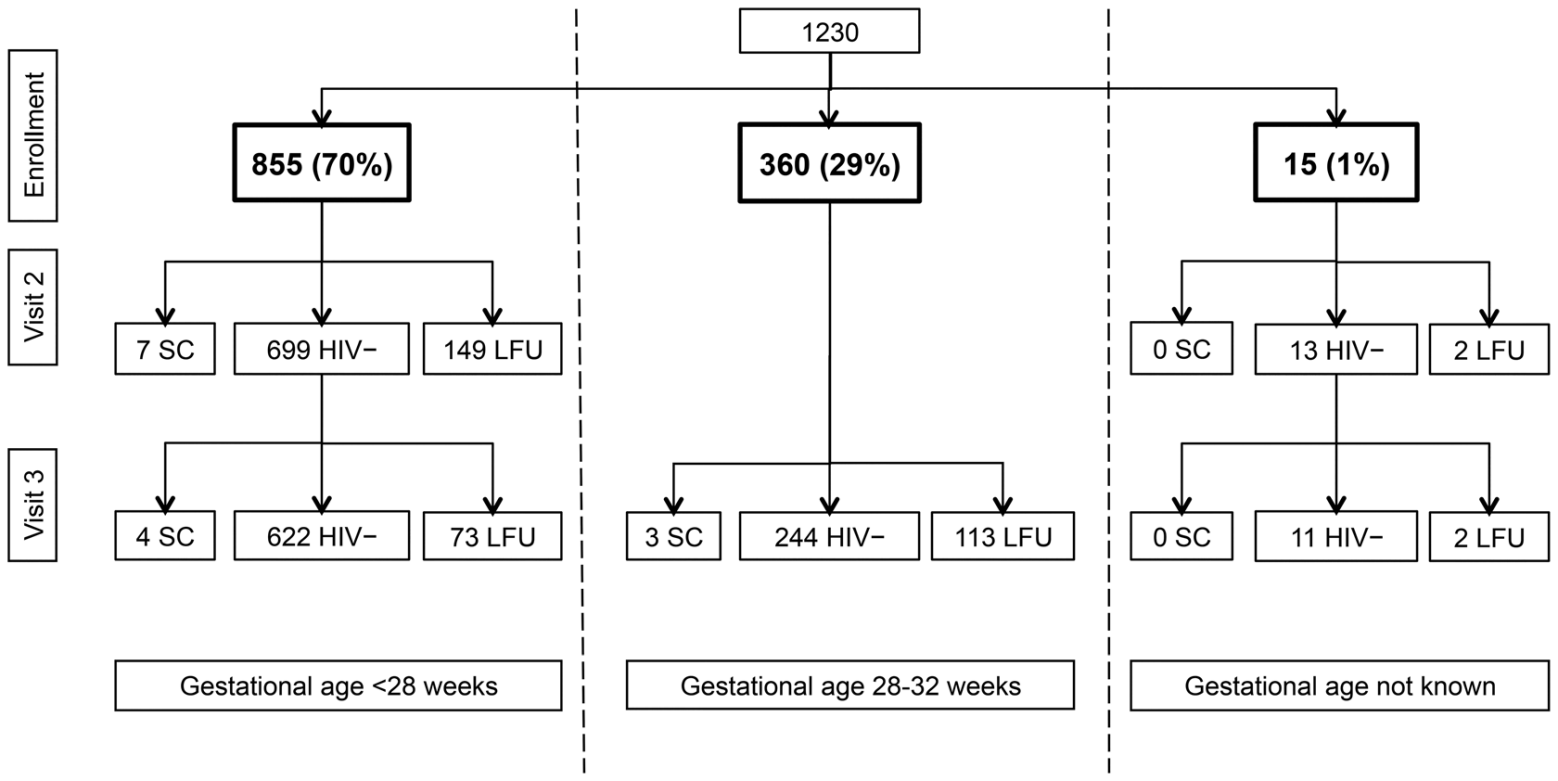
Flowchart of the cohort study. Study visits were scheduled at enrollment, 28 weeks of gestational age (visit 2), and delivery (visit 3). Women enrolled at gestational age <28 weeks (n = 855) had three scheduled visits. Women who enrolled between 28 and 32 weeks of gestational age (n = 360) participated in only two of the three study visits (enrollment visit and delivery). Fifteen women enrolled at a gestational age between 0–32 weeks, but exact age was not known. SC seroconversion; HIV – HIV negative; LFU lost to follow-up.

A total of 1230 pregnant women were enrolled in the cohort with 151 (12%) lost to follow-up (LFU) after the enrollment visit and an additional 188 (15%) as they missed their last study visit at delivery (Fig. 1). The median follow up time for 968 women who had at least one follow up visit was 124 days (IQR 76–141). Baseline characteristics of the cohort are shown in Table 1. The median age at enrollment was 24 years (IQR 20–29) with a median gestational age of 24 weeks (IQR 21–28). This was the first pregnancy for 196 (16%) women.

**Table 1:** Baseline characteristics of the study population (n = 1230).

		Total (n)	Percentage/median (IQR)
		1230	
**Province**			
	Maputo	591	48%
	Gaza	639	52%
**Age (years)**		1201	24 (20–29)
	Missing	29	
**First pregnancy/subsequent pregnancy**			
	First pregnancy	196	16%
	Subsequent pregnancy	971	79%
	Missing	63	5%
**Gestational age at inclusion (weeks)**			
	10–23 weeks	431	35%
	24–32 weeks	784	64%
	Missing	15	1%
**Parity**			
	<3	763	62%
	≥3	404	33%
	Missing	63	5%
**Age at first child (years)**			
	Median (IQR)	1123	18 (17–20)
	Missing	107	
**Marital status**			
	Married/living with partner	820	67%
	Relationship but not living with partner	132	11%
	Divorced/separated/widow/single	275	22%
	Missing	3	0%
**Polygamous marriage (out of the married couples)**			
	No	632	77%
	Yes	127	15%
	Missing	61	7%
**Educational level**			
	<Primary education	819	67%
	Complete primary education or more	400	33%
	Missing	11	1%
**Partners' educational level**			
	<Primary education	537	44%
	Complete primary education or more	482	41%
	Missing	211	17%
**Knowledge if partner was ever tested for HIV, at baseline**			
	Not tested	685	56%
	Tested and HIV negative	205	17%
	Tested and HIV positive	8	1%
	Tested but don't know result	16	1%
	Don't know if tested	232	19%
	Missing	84	7%
**Condom use ever with the current partner**			
	Never	974	79%
	Sometimes	225	18%
	Always	9	1%
	Missing	22	2%
**Ever had extramarital sexual activity before enrollment**			
	No	1162	95%
	Yes	27	2%
	Missing	41	3%
**Knowledge if partner ever had extramarital sexual activity before enrollment**			
	No	450	36%
	Yes	192	16%
	Don't know	588	48%

At enrollment, 229 (19%) of the women reported that their partner was tested for HIV, 685 (56%) reported that their partner was *not* tested for HIV and 232 (19%) reported not knowing if their partner was ever tested. Of the 229 women who reported to know that their partner was ever tested for HIV, 213 (93%) reported to know the test result. Eight of them said their partner was HIV positive. There was no difference among women with and without follow-up visits. Reported serodiscordancy (male+/female−) at enrollment was 4% (8/213). Among those eight discordant couples, five women (63%) reported never using condoms with their partner, two (25%) reported that they sometimes do and one (12%) reported that they always do. Overall, 79% of women reported that they never used a condom with their partner.

During the study follow-up, 62 (9%) partners of women who reported that their partner was not previously tested, received HIV testing, while 76 (37%) partners of those who reported that their partner was tested and HIV negative, were retested. In both groups, none of the partners tested positive. Similarly, 25 (11%) partners of women who did not know if their partners had been tested were subsequently tested, with a positive HIV test result in two (8%). Information about sexual activity during the study period was provided by 494 women: 220 women reported not having had sexual activity with their partner, while 274 reported having had sex with their partner. Extramarital sexual activity during pregnancy was reported by 80 women, of whom 2 declared to have had sexual activity outside the marriage during the study period. Among participants who responded about partner extramarital sexual activity, 35% (77/221) of their partners had extramarital sexual activity during the study period.

Women who were lost from the study after enrollment visit were more likely than women with study follow-up to be from Gaza Province (72% versus 46%; p = 0.0001), have a gestational age >23 weeks at enrollment (73% versus 61%; p = 0.001), be single (38% vs 18%; p<0.0001), have less than complete primary education (74% versus 65%; p = 0.001) and have a partner with less education (50% versus 42%; p = 0.008).

### HIV incidence and associated risk factors

During the follow up period, 14 new HIV infections were detected by HIV rapid testing, seven at the second study visit and seven at time of delivery. On confirmatory HIV DNA-PCR testing, all of the samples of the time of seroconversion were positive. Eleven out of the 13 cases were found to be DNA-PCR positive at enrollment; one sample of the enrollment visit was missing.

Total follow up was 328 women years. HIV incidence in the cohort is calculated at 4.28 per 100 women-years (95% CI: 2.33–7.16). Only one woman with seroconversion had a partner known to be HIV positive at enrollment; five partners were reported to be HIV negative and eight partners were of unknown serostatus. Only one partner of a newly infected woman was reported to have been retested during study follow-up (HIV negative), one partner was known not to be retested and in 11 it was not known if retested.

Analysis of potential risk factors associated with HIV seroconversion in univariate analyses are shown in Table 2. The only significant associations seen were a 4-fold increased risk among women living in Maputo (p = 0.05) and women with sexual debut before 17 years of age (p = 0.04).

**Table 2:** Risk ratios for socio-demographic, sexual behavior and knowledge risk factors among women with follow-up (n = 968).

		Number	Number SC	Risk Ratio (95% CI)	P-value^1^
**1. SOCIODEMOGRAPHIC RISK FACTORS**					
**Province**					
	Maputo	518	12	4.35 (0.97–19.45)	**0.05**
	Gaza	450	2	Reference	
**Age (years)**		942		0.94 (0.83–1.05)^2^	0.28^2^
**First/Subsequent pregnancy**					
	First pregnancy	163	4	Reference	
	Subsequent pregnancy	757	10	0.62 (0.19–1.98)	0.42
**Gestational age at enrollment**					
	10–23 weeks	361	3	Reference	
	24–32 weeks	594	11	3.25 (0.91–11.64)	0.07
**Marital Status**					
	Married/living with partner	677	11	Reference	
	Relationship but not living with partner	114	2	1.02 (0.23–4.59)	0.98
	Divorced/separated/widow/single	175	1	0.32 (0.04–2.44)	0.27
**Polygamous marriage (out of the married couples)**					
	No	519	6	Reference	
	Yes	106	2	1.78 (0.36–8.82)	0.48
**Educational level**					
	<Primary education	625	8	Reference	
	Complete primary education or more	339	6	1.28 (0.44–3.68)	0.65
**Partner Educational level**					
	<Primary education	406	2	Reference	
	Complete primary education or more	397	9	4.09 (0.88–18.94)	0.07
**Employment status participant**					
	No	750	10	Reference	
	Yes	68	2	2.38 (0.52–10.88)	0.26
**Employment partner**					
	No job	181	1	Reference	
	Employee	174	5	4.67 (0.55–4.00)	0.16
	Own business/sales	132	2	2.62 (0.24–28.93)	0.43
	Mineworker	242	3	2.48 (0.26–23.73)	0.43
	Other	229	3	2.22 (0.23–21.44)	0.49
**Absence of partner at least one continuous month**					
	No	594	11	Reference	
	Yes	336	3	0.55 (0.15–1.97)	0.36
**2. SEXUAL BEHAVIOR RISK FACTORS**					
**Age at first sexual activity (years)**					
	< = 16 years	406	10	3.79 (1.04–13.78)	**0.04**
	17–27 years	422	3	Reference	
**Ever tested for HIV prior to this pregnancy**					
	No	507	7	Reference	
	Yes	397	7	1.34 (0.47–3.82)	0.55
**Knowledge of partners' HIV status at baseline**					
	Do not know partners' status	177	6	Reference	
	Know partners' status	729	8	2.82 (0.98–8.13)	0.055
**Serodiscordancy at baseline**					
	Partner HIV negative	171	5	Reference	
	Partner HIV positive	6	1	3.85 (0.45–32.99)	0.22
**Condom use ever with the current partner**					
	Never	761	12	Reference	
	Sometimes	186	1	0.29 (0.04–2.22)	0.23
	Always	9	1	8.10 (1.05–62.31)	**0.04**
**Ever had extramarital sexual activity before enrollment**					
	No	926	13	Reference	
	Yes	19	1	3.94 (0.52–30.12)	0.19
**Knowledge if partner ever had extramarital sexual activity before enrollment**					
	No, he had not	322	2	Reference	
	Yes, he had	159	0	3.3 e-8 (0-)	>0.999
	Don't know if he had	487	12	3.28 (0.73–14.64)	0.12
**3. KNOWLEDGE RISK FACTORS**					
**Identification of VT risk during pregnancy**					
	No	328	7	Reference	
	Yes	538	6	0.57 (0.19–1.71)	0.32
**Identification of VT risk during delivery**					
	No	451	9	Reference	
	Yes	415	4	0.53 (0.16–1.73)	0.29
**Identification of VT risk during breastfeeding**					
	No	162	2	Reference	
	Yes	704	11	1.12 (0.25–5.05)	0.88
**Is there cure for HIV**					
	No	678	12	Reference	
	Yes	124	1	0.51 (0.07–3.91)	0.52
	Don't know or no response	102	1	0.51 (0.07–3.96)	0.52

SC =  seroconversion; VT =  vertical transmission.

1 p-values from bivariable Poisson regression models (i.e., generalized linear models using the Poisson distribution with the log link and an offset accounting for person-years).

2 Risk ratio for a age compares two groups that differ by one year.

Boldface  =  statistically significant (p<0.05).

There was a marginally significant higher HIV incidence rate among women who knew their partners' HIV status compared to those who did not know their partners' HIV status (p = 0.055). Of note, 171 reported that their partner was HIV negative and 6 partners were HIV positive. Trends were also seen in higher risk for women with more educated partners (RR 4.09 [95% CI: 0.88–18.94], p = 0.07) and women with higher gestational age at enrollment (RR 3.25 [95% CI: 0.91–11.64], p = 0.07).

### Motivators and Barriers to male partner HIV testing

Between August and November 2011, individual interviews were conducted in the selected study facilities with 15 men, 10 women and 12 nurses. Additionally, 11 FGD were held: three with 22 men, four with 35 women, and four with 36 grandmothers. One woman with a three year old child participated in a FGD although she was outside of the eligible postpartum period. Characteristics are available for all but the HCW (Table 3) and a summary of barriers and motivators are presented in Table 4.

**Table 3:** Characteristics of participants of the qualitative study (n = 118).

		Men	Women	Grandmothers	Total
		37	45	36	118
**Age (years, median, IQR)**		42 (30–56)	26 (22–32)	55 (50–60)	
**Educational level**					
	<Primary education	24	24	34	82
	Complete primary education or more	13	21	2	36
**Marital status**					
	Married/Living with partner	35	30	20	85
	Divorced/separated/widow/single	2	15	16	27
**Parity**					
	<3	19	21	8	48
	≥3	18	24	28	70
**Age of youngest child (years, median (IQR) (for parents only)**					
		4 (1.3–8)	0.9 (0.3–1.8)		
**Pregnancy (for women only)**					
	Not pregnant		42		
	Pregnant		3		

**Table 4:** Summary of barriers, motivators of accessing HIV testing by male partners.

	Barriers	Motivators
**Knowledge**	- Assume test result of partner or child is the same so no need to go for HIV testing	- You can't assume partner or child result
		- Knowing the importance of getting tested (wanting to know status) and treated early
**Perceptions**	- Health facility is a place for women only	- Health facility is the right place for HIV testing and care
**Behavior/attitude**	- Fear of stigma and discrimination	- Family and community support
	- Fear of disclosure	- Would go to health facility if invitation was given
	- Cultural taboos/rituals	
**Other**	- Absence (migrant worker)	- Priority for couples at MCH services
	- Men don't have time	
	- Services not male-friendly	

*MCH – Mother and Child Health.*

#### a. Barriers of uptake of HIV testing

One of the most important barriers is fear of discrimination or stigma, which was reported by all groups except HCW. During the interviews and FGD, the fear of *women* losing their home was mentioned frequently as a barrier for women to test, disclose or invite their husbands for testing. Men and women said that men don't want to get tested for fear of a positive test result.

*“It can also happen that he is scared to be seen by somebody in the health center who will go and spread in the area where he lives that he is in that situation (meaning being HIV positive)” (Focus Group Discussion, Moamba, Men)*

One barrier related to knowledge about HIV testing that was mentioned by some women is that male partners assume they have the same test result as their female partner (in this case the pregnant woman) or child, which makes it unnecessary for them to go for HIV testing.

*“Sometimes, you can go and do the test, and they say you are negative; you come to say to him you did the test and is negative, he says: so I am also negative, I don't need to go there any more” (Focus Group Discussion, Boane, Women)*

Some HCW indicated that the MCH environment is not male friendly and not conducive to attending to men or couples. Some men felt that women are offered various (MCH) services while there is no motivation/reason for men to go, considering those services as a “place for women”. Cultural taboos or rituals cause people/men not to go for HIV testing to a health facility, such as men not being allowed to see women's blood. This was mentioned by most HCW.

*“Taboo such as for example the man that cannot see the woman's blood, so she can't go with her husband to the hospital; only mothers-in-law go who are also women”(Individual Interview, Moamba, HCW)*

Other barriers include distance to the health facility or the partner being absent (working as a migrant worker in South-Africa). Men indicated that they don't have time.

#### b. Motivators of uptake of HIV testing

All groups except HCW said that men (and women) are motivated to get tested because they know the importance of getting tested and starting treatment early if needed.

*“People do the test, because they want to know about their health” (Individual Interview, Chicumbane, Women)*

In contrast to above, about half of the men and women knew that it is necessary to get tested for themselves, as they cannot assume their results are the same as their partners' or infants'. Some of the men said that they would accept an invitation to go for HIV testing if it was given to them.

Grandmothers indicated that the health facility is the right place to go for HIV testing and care. They frequently mentioned that support from the family (in most cases themselves giving support to a son and daughter-in-law) and community helps men to adhere to HIV testing services.

*“Because she will be scared to tell her husband, say it to me and I will join them and say to my son that your wife delivered and had this and that, and go with your wife to get examined so you can get similar medication as your wife.” (Focus Group Discussion, Chibuto, Grandmothers)*

HCW noted that men and couples get priority at MCH services, and the resulting decrease in waiting time is a facilitator for male HIV testing.

Participants recommended more counseling and testing at the community level, including “health campaigns”, home based counseling and testing, sensitization sessions directed at men, and strong involvement of community leaders and community health workers. At the health facility, a more men-friendly approach was suggested by some men, women and HCW: creation of a men-friendly room at MCH, flexible opening hours to accommodate men's working hours and invitations sent out by the health facilities.

## Discussion

This study identified a high HIV incidence rate (4.28 per 100 women-years) in a population of pregnant women in Southern Mozambique, similar to other countries in the region [11], [13]–[17]. This incidence during pregnancy is higher than that seen in a cohort followed in the same facilities from delivery to 18 months postpartum (3.2 per 100 women-years) [10]. However, a recent meta-analysis on HIV incidence during pregnancy and postpartum did not show significant differences in the pooled incidence rates during pregnancy compared to postpartum period (4.7 per 100 women-years during pregnancy versus 2.9 per 100 women-years during the postpartum period) [2]. This cohort of pregnant women in Southern Mozambique had similar high incidence rates to sexually active women in the southern region (4.6 per 100 women-years) and slightly lower than in the central region in a study involving women with multiple sexual partners in the last month (6.5 per 100 women-years) [18], [19]. In these settings, major investments should be directed at primary prevention strategies for all women in reproductive age.

The HPTN052 study, conducted among stable discordant couples, demonstrated the importance of identifying discordant couples and early initiation of ART to prevent transmission within the couple [20]. However, it also found that 18% of the HIV transmissions were not linked to the partner [20], indicating that focusing efforts on the partner alone may not be sufficient to address the challenge of seroincidence in pregnancy. Besides the possible biological reasons for a higher susceptibility among pregnant women, risk behaviors should not be forgotten as an important factor [2], [13]. Our study results showed that some women reported extramarital sexual activity during pregnancy. Increased HIV risk behaviors during pregnancy have been seen for example in Mpumalanga, South Africa, where 20% of pregnant women declared having at least 2 sexual partners in the last 3 months [21]. Studies on risk behavior among partners of pregnant women are scarce. Our findings show that 35% of the women reported that their partner had an extramarital sexual relationship during the study period. This is higher as in the neighbouring country South-Africa where 18% of the male partners had sex with somebody else than the pregnant partner in the previous month [22].

Partner testing remains extremely low in sub-Saharan Africa, usually between 10 and 16% [7], [23], [24]. Our study revealed that 19% of the pregnant women reported to know that their partner was ever tested, slightly higher than those reports. Not testing among couples increases the chance of unknown serodiscordancy, with a higher risk of HIV acquisition and vertical transmission. The Mozambique national HIV survey showed a serodiscordant rate of 10% with half being male positive discordance [9], which is a similar result to what we found among pregnant couples (4%). Knowing the partners' HIV status resulted in a higher risk of seroconversion in our study, even though the majority reported HIV negative partners. This could be due to reporting bias, unlinked seroincident infections in the partner with subsequent transmission to the pregnant women, or a false feeling of security when the couple receives an initial negative HIV result.

The barriers to partner testing identified in the qualitative study point to several alternative strategies to increase HIV testing among partners. Priority is given to men when accompanying their partners to MCH, however this has not resulted in significant testing. Initiatives to attract men to health facilities by creating more men-friendly services, or provision of mobile health prevention services for men are needed. Another suggestion from participants was to move the counseling and testing to the community. Community testing is part of the Mozambique Ministry of Health's program, but it does not focus on pregnant women and their families.

Expansion of health prevention and care services to the community could play a critical role in improving access to reproductive health care services for men, women, adolescents and couples. Home based couple testing has been seen as acceptable but issues such as stigma and disclosure need to be addressed, and counselors should be appropriately trained [25]. A study done in Uganda suggests an increase in HIV testing when done at the households [26] and success has been seen with community intervention packages with a couple centered approach for MCH/PMTCT services [27], [28]. A recent review showed that non-health facility initiatives lead to higher male involvement than facility based initiatives [29]. Other primary prevention activities directed to men at workplace, including mining companies, should be strengthened as they might be more accessed by men than primary health care services.

The strength of our study is that seroconversion was measured within a cohort study, giving the most reliable information on new HIV infections. Limitations include a high loss to follow-up, which limited multivariate and longitudinal analyses. Women lost after enrollment differed from those followed in some characteristics, which may have led to underestimated incidence results (e.g. province and partner level of education). This was an operational research study where women were tested for HIV using the national algorithm of rapid assays in all study visits. The operational nature of the study did not permit the testing of all enrolled women with a molecular assay. For study purposes, stored DBS specimens from women who seroconverted to positive on standard rapid HIV testing were tested using HIV DNA PCR (13 tested out of 14 seroconverters). We found that 10 of these 13 women with a negative HIV rapid assay on the initial visit were positive on DNA-PCR testing. Conversely, it is also possible that women with a negative HIV rapid test on the last study visit were infected with HIV but still undetected with an antibody assay. Since the difference in the diagnostic window between rapid antibody and molecular assays are usually around 7–10 days and as detected seroconversions were equally distributed across the study visits, it is plausible that true new infections were equally prevalent, with little impact on the incidence rates described here. Therefore, we used seroconversion as a proxy for seroincidence. As in all surveys, there is a risk of reporting bias on information given regarding individual and partner's sexual behavior. Data on vertical transmission were not available for women in this study.

HIV incidence during pregnancy is high and partner HIV testing is low in Southern Mozambique, emphasizing the urgent need for increased support for primary prevention within MCH services. Reaching men for HIV testing, prevention and/or treatment is crucial. Knowledge of both partners' HIV status is critical for maximum effectiveness of prevention and treatment services to reach elimination of pediatric HIV/AIDS.
